# The Crisis of the Voluntary Hospital

**Published:** 1892-07-30

**Authors:** 


### The Hospital

AN INSTITUTIONAL JOURNAL OF

**Science, Medicine, Nursing, and Philanthropy.**

*For Hospitals, Asylums, Infirmaries, Dispensaries, Medical Practitioners, Students, Nurses, and the Charitable Public.*

VOL. XII.—No. 305.] [JULY 30, 1892.

### THE CRISIS OF THE VOLUNTARY HOSPITALS.

THERE can be no question that a crisis is approaching in the history of the voluntary hospitals of this country. On the wise solution of this most vital controversy will depend not only the well-being, but possibly the very existence of our hospitals as at present constituted. It is admitted by all, or nearly all, the most representative of hospital managers that unless something effective can be accomplished whereby co-operation and combined action is secured in the interests of all the institutions, whilst preserving and emphasizing the individual character of our charities, outsiders will lay their hands upon the edifice, and disaster must follow. There is an uneasy and restless spirit abroad. The policy of making Englishmen more and more to resemble the jelly fish, and less and less the man of independent action, seems to grow in popular favour. The cry for State subsidies, and rate support is more and more met with every day. So there is an increasing cry for all hospitals to be rate supported. In such circumstances it is no use wringing hands, or closing eyes, for the crisis is upon us, and has to be faced with an united and sustained effort.

All well-informed people are agreed that the voluntary principle has made our hospitals the best administered in the world. Despite this, or possibly because of it, the hungry, restless placeman of our political era has begun to throw hungry glances at the hospitals, and it may not be long before he strives to take possession. Should he be allowed to succeed in his efforts, the suffering poor will experience an injury which will redound to the discredit of our day and generation. Further, such a success will indelibly prove that we are made of baser stuff than our sires, and so England's greatness will give place in due time to a decadence which may prove the ruin of our country. It is a noble and glorious privilege we Englishmen possess of showing to the civilised world how to adapt Christianity to the most practical movements of everyday life. To succour the sick and suffering, feed the hungry, clothe the naked under a system based upon and maintained entirely by the voluntary and sustained efforts of individual citizens is an achievement of which any nation might be proud. England, the mother of nations, the home of a free people, the centre of the markets of the world, has accomplished this after centuries of devoted effort on the part of some of the noblest of her sons. Are we, the men of to-day, going to permit any political party or any spurious outcry on the part of restless busybodies, however numerous and persistent, to destroy the noble edifice of voluntary effort, and to substitute for it a dry, perfunctory, one-sided organisation, which would speedily degenerate into a mere "good enough" inefficiency, the horrors of which can be seen by the curious in some of the metropolitan and provincial workhouses of to day, and, unless report is wrong in certain of the fever hospitals of the Metropolitan Asylums Board also? The crisis is upon us, then, and it becomes hospital managers to meet it like men, and to save the country from the disgrace, and the suffering poor from the miseries, which may follow any failure to solve it wisely.

The meeting at Spencer House on Friday, over which the Lord Mayor presided—the present Lord Mayor has surpassed all previous holders of his office in the efforts he has made for the hospitals, a fact worthy of the most grateful remembrance and recognition—was attended by representatives of the principal general hospitals having medical schools. If the discussion may be taken as an index of the facts, those present were authorised to represent the various hospitals, and did not attend merely as individuals interested in the subject. The decision to appoint a small Committee to consider the Lords' suggestions, and to report to a future meeting of the Conference may, therefore, be taken as representing a general desire on the part of the hospitals to give the most candid consideration to the recommendations in question. It was noticeable, however, that there was, on the whole, a want of genuine interest in the proposals made, an absence of any solid belief in the practicability of devising a scheme of co-operation which could be made generally acceptable, and beyond this, an undercurrent of desire to do nothing. If our judgment on these points be correct there is much to regret, and we would earnestly urge the various Hospital Committees to take up the Lords' Committee's Report, to go carefully through the points raised, and to come to a decision upon each of them. By adopting this course they will arrive at a clear perception of the position it is desirable for them to take up in their own interests, and in that of all the hospitals. Then, when the Conference meets again to consider the Report and recommendations of the Special Committee, the members who attend it will be prepared to take up a definite position, and to sustain it by arguments which must carry due weight. There is a general desire to show the utmost respect to the Lords Committee, which has exhibited remarkable restraint in preparing the exhaustive summary of the evidence given during three sessions of Parliament. The Lords' Committee were strongly of opinion that some central voluntary board exercising supervision and control over all medical relief agencies throughout London would tend to diminish abuse and increase public confidence. Controversy is likely to arise not so much around this proposal *per se*, which is generally assented to, but on the details of such a board's constitution and *personnel*. It would be best, perhaps, to proceed tentatively at first by utilizing the existing agencies, and so to pave the way for a separate central voluntary board when greater experience has been gained of the nature of the work likely to devolve upon its members.

The Committee appointed on 22nd inst. will have a somewhat thorny path to traverse if they go over all the ground covered by the Lords' inquiry. It will behove them to consider at the outset whether it will not be wiser to confine their attention to the proposal to appoint a Central Board without statutory power. Such a Board not possessing any authority to enforce its recommendations, and being without the power of the purse at its back, would probably accomplish little, whilst its powers for evil might be considerable. The voluntary hospitals not unnaturally object to be taxed in order to defray the cost of setting up a Board they do not greatly desire, and which might be the means of doing many things to which they have solid objection.

If any Central Board is to be formed, and many hospitals have no objection to such an authority, it must be so constituted as to possess a representative character, and at the same time be in a position to enforce its decrees and to maintain itself without cost to the existing hospitals. We have at the present time several organizations of a more or less representative character, which might be made the basis for such a Central Board. We can only hope the Special Committee will give very careful consideration to this aspect of the question, and that meanwhile the various hospital committees will consider to what extent they are prepared to co-operate with other institutions in forming a Central Board, with the object of defeating criticism, increasing the income of all the voluntary hospitals, and providing a ready and effectual means of preventing the undue multiplication of so-called hospitals which at present deceive the public and the poor, whilst they cause incalculable injury to all reputable institutions.

